# Degradation Kinetics of Anthocyanins from European Cranberrybush (*Viburnum opulus L.*) Fruit Extracts. Effects of Temperature, pH and Storage Solven

**DOI:** 10.3390/molecules171011655

**Published:** 2012-09-28

**Authors:** Bianca Moldovan, Luminiţa David, Cristian Chişbora, Claudia Cimpoiu

**Affiliations:** Faculty of Chemistry and Chemical Engineering, Babeş-Bolyai University, 11 Arany Janos Str., 400028, Cluj-Napoca, Romania

**Keywords:** anthocyanins, cranberrybush (*Viburnum opulus* L.) fruits, stability, degradation kinetics

## Abstract

European cranberrybush (*Viburnum opulus* L.) fruits are well known for their biological properties, of which some are due to the presence of anthocyanins in the berries. Current literature provides little information concerning these fruits. The stability of anthocyanins from *Viburnum opulus* fruits, in aqueous and ethanolic extracts, stored under darkness for 7 days at different temperatures (2 °C, 37 °C and 75 °C) and pH values (pH = 3 and 7), was studied here. The lowest stability was showed by the anthocyanins from the water extract stored at 75 °C and pH = 7, with half-life and constant rate values of 1.98 h and 0.3488 h^−1^, respectively. The results showed a good correlation between the total anthocyanin content (determined using the pH differential method) and the time of storage, with determination coefficients varying from R^2^ = 0.9298 to R^2^ = 0.9971. Results indicate that the storage degradation of anthocyanins followed first-order reaction kinetics under all investigated conditions.

## 1. Introduction

European cranberrybush (*Viburnum opulus L.*—also known as Snowball tree, Guelder rose or Crampbark) belongs to the *Caprifoliaceae* family. This plant is widely distributed especially in the Eastern Europe countries, Turkey, North Asia and North Africa and it is generally used as an ornamental plant. The cranberrybush has a red fruit, ripened in August–September, which remains through the winter. The berries are edible, although seldom consumed directly as food as they are bitter and highly astringent [1]. Among the dietary usages, the juice from the berries is the best known product; however, the berries can also be cooked into preserves like jams, jellies, marmalades or fermented to make an alcoholic drink. The berries are also well known for their biological properties, traditionally being used for the treatment of menstrual, stomach and kidney cramps, duodenal ulcers, high blood pressure, heart troubles, coughs and colds [2,3,4].

Reported studies on cranberrybush fruits and their properties are limited. Given their known health benefits, the content in anthocyanin for berries of some *Caprifoliaceae* species has lately received attention [5,6]. The European cranberrybush berries are remarkable because of their elevated antioxidant activity, which is due to their very high content of polyphenolic compounds [7].

**Scheme 1:**
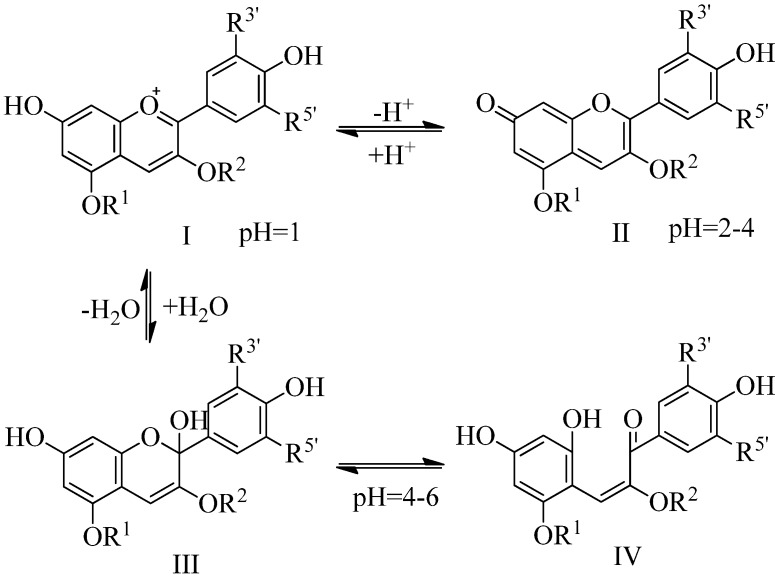
Variation of anthocyanins’ chemical structures at different pH values.

Anthocyanins are a group of naturally occurring phenolic compounds, which play an important role in the color quality of many flowers, fruits, vegetables and related products derived from them. Recently, anthocyanins were reported to have important biological activity, presenting antioxidant, antimutagenic, anticancer and antiobesity properties [8,9,10], as well as reducing the risk of coronary heart disease [11]. Besides their biological activities, the bright colour of anthocyanins (orange, red, purple, blue), ensures a high potential of being used as natural dyes as an alternative to artificial colorants. The colour of anthocyanins depends essentially on the chemical structure of different forms in which they can be found, these structures being strongly related to the pH value of the solution. For example, the flavilium cation I (red colour) is the predominant species at pH = 1, while at pH values between 2 and 4, the quinoidal blue base II predominates (Scheme 1). 

As the pH is raised up to 6, two colorless carbinol pseudobases III are formed, which can undergo ring opening to a yellow chalcone IV [12]. Under basic conditions, a degradation of the anthocyanins occurs. At a pH typical for fresh and processed fruits and vegetables, between 4 and 6, a mixture of equilibrium forms: flavilium cation, anhydrous quinoidal base, carbinol base and chalcone coexists. However, due to their high reactivity, anthocyanins easily convert to colorless or undesirable brown degradation compounds. Among many factors that can influence anthocyanin stability, the most significant is temperature. Anthocyanins present a very high thermal sensitivity [13]. Besides the temperature, pH, light, oxygen, enzymes, ascorbic acid, sugars and hydrogen peroxide also affect the stability of anthocyanins [14,15,16,17]. Thus, measurement of anthocyanin content and investigation of their degradation is useful for the food industry. However, to date, no information is available in the literature on the degradation kinetics of anthocyanins found in European cranberrybush fruits.

In order to predict the quality changes of anthocyanins during their storage and processing, the accurate determination of the degradation kinetic parameters is a matter of a great concern. The purpose of this study was to determine the storage and thermal stability of the anthocyanins from European cranberrybush fruits extracts in different storage solvents at different temperatures and pH values. Accurate knowledge of the degradation kinetics for the anthocyanins is essential for predicting changes that may occur either during storage in various conditions or/and during thermal processing of food products (especially fruit juices) containing these anthocyanins. The conditions investigated here assure a high compatibility with storage (refrigerated and room temperature storage) and processing techniques generally applied in the food industry (for example, 75 °C representing juice pasteurization temperature).

## 2. Results and Discussion

### 2.1. Total Anthocyanins Content

The content of anthocyanins in European cranberrybush fruits was calculated to be 0.356 ± 0.014 g/kg (frozen fruit), expressed as cyanidin-3-glucoside. This content was higher than the value found in European cranberry bush by Deineka *et al*. [18], who observed a total anthocyanin content of 0.22–0.29 g/kg fresh fruit. The berries we investigated have considerably lower anthocyanin content compared to other fruits from *Caprifoliaceae *family, like black elderberry, which are particularly rich in anthocyanins (0.42–0.86 g/100 g fresh fruit) [18]. Our values of total anthocyanin content are also in general lower compared to other different common anthocyanin rich fruits, like chokeberry (560 mg/100 g fresh weight) [19], bilberry (300–320 mg/100 g fresh weight), blueberry (83–326 mg/100 g fresh weight) [20], but higher than the value found in strawberry (15–35 mg/100 g fresh fruit) [21] and red raspberry (20–60 mg/100 g fresh fruit) [20].

### 2.2. Degradation Kinetics of Anthocyanins during Storage

Thermal degradation of aqueous and ethanolic extracts of anthocyanins from European cranberrybush fruits was studied at various pH values (3 and 7) and at different temperatures (2 °C, 37 °C and 75 °C).

The degradation of monomeric anthocyanins from *Viburnum opulus* fruits was studied in aqueous and ethanolic extracts during storage at 2 °C, 37 °C and 75 °C.The determined values for the kinetic rate constants and the half-life values are summarized in Table 1.

**Table 1:** Kinetic parameters of degradation of anthocyanins from Cranberrybush fruits extracts in different conditions.

pH	Temp. (°C)	Solvent	k × 10^3^ (h^−1^) ^a^	t_1/2_ (h)
3	2	Water	0.6 (0.9503)	1,155
3	37	Water	6.4 (0.9740)	108.28
3	75	Water	46.1 (0.9971)	15.03
7	2	Water	2.8 (0.9680)	247.5
7	37	Water	30.5 (0.9944)	22.72
7	75	Water	348.8 (0.9762)	1.98
3	2	Ethanol/Water	1.3 (0.9575)	533.07
3	37	Ethanol/Water	13.6 (0.9602)	50.95
3	75	Ethanol/Water	53.9 (0.9948)	12.86
7	2	Ethanol/Water	2.5 (0.9298)	277.2
7	37	Ethanol/Water	38.5 (0.9867)	18.0
7	75	Ethanol/Water	189.3 (0.9824)	3.66

^a^ Numbers in parantheses, R^2^, are the determination coefficients.

**Figure 1:**
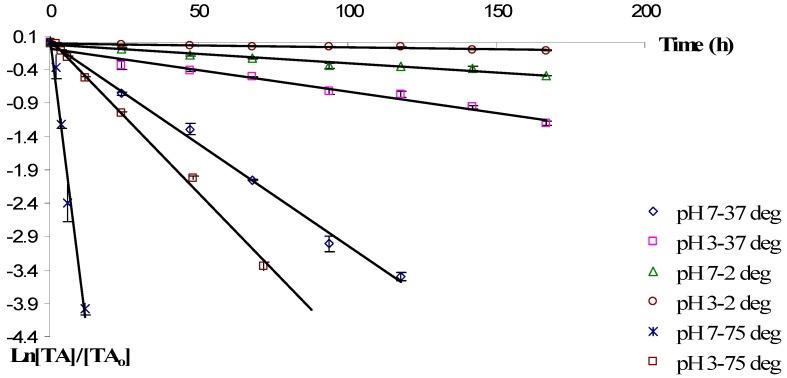
Degradation of anthocyanins in cranberrybush fruits aqueous extract during storage at different temperature and pH values (Vertical lines represent SD, n = 3).

The contents of aqueous anthocyanins from *Viburnum opulus* *(L.)* fruits during storage were plotted as a function of time (Figure 1). The linear regression of the total anthocyanins content during storage confirmed that degradation of aqueous anthocyanins from European cranberrybush (as observed in Figure 1) followed first order reaction kinetics. The obtained results are in agreement with those from the previous studies which showed that storage degradation of anthocyanins from various sources is described by first order reaction kinetics [22,23,24]. The kinetics for this reaction type can be expressed by the following equations:

ln[TA] = ln[TA_0_] − kt (1)

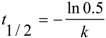
(2)

where [TA] = total anthocyanin content (mg/L) at t = time; [TA_0_] = initial total anthocyanin content (mg/L); k = reaction rate constant (h^−1^); t = reaction time (h); t_1/2_ = half-life (h).

The rate constants and the half-life values (Table 1) indicate a significant influence of the pH of the storage solution on the stability of anthocyanins which correlate to the dependence of their structure on pH. In agreement with other studies showing the influence of the pH value on the stability of anthocyanins [25], we observed that acidic media increased the stability of anthocyanins from aqueous *Viburnum opulus* *(L.)* fruits extract, as indicated by higher t_1/2_ values.

By comparing the half-life values, one can conclude that, at lower temperatures, European cranberrybush anthocyanins in aqueous extract are ~4.7 times less susceptible to degradation than they are at lower pH values (t_1/2, pH = 3_/t_1/2, pH = 7_ ≈ 4.7), the highest stabilities being observed at pH = 3, regardless of storage temperature. At higher temperatures, such as 75 °C, the effect of pH on the degradation process becames more significant, the stability of anthocyanins at pH = 3 being 7.6 fold higher than at pH = 7. 

The thermal stability of the extracts was also evaluated. As expected, the degradation rate of anthocyanins increased with the increase of temperature. Regardless of the pH value, storage at 37 °C resulted in a 10.7 times faster degradation as compared to degradation at refrigerated storage (at 2 °C). At pH = 7, the effect of temperature on the degradation process is more important. At lower pH = 3, the degradation process at 75 °C occurs 76.8 times faster than at 2 °C, while at higher pH (pH = 7), the ratio t_1/2, 2 °C_/t_1/2, 75 °C_ is 125 in aqueous extract.

**Figure 2:**
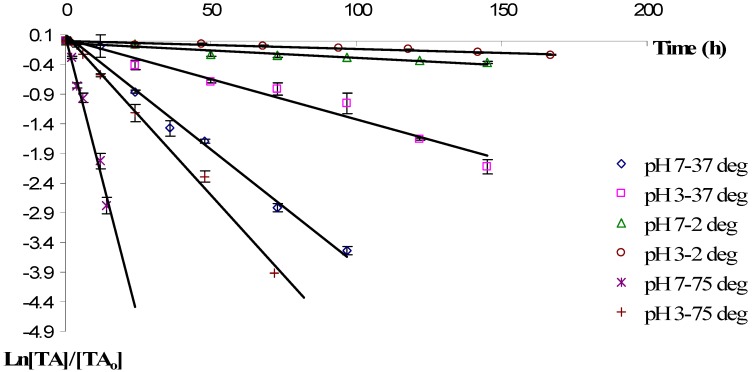
Degradation of anthocyanins in cranberrybush fruits ethanolic extract during storage at different temperature and pH values (Vertical lines represent SD, n = 3).

The degradation of monomeric anthocyanins from European cranberrybush fruits in ethanolic extract was also investigated at the same pH and temperature values as the aqueous one. The total content of anthocyanins from ethanolic extract of *Viburnum opulus (L.)* fruits during storage was plotted as a function of time (Figure 2). In all investigated cases, the degradation was fitted to a first order reaction model.

As observed in the case of aqueous solutions, the European cranberrybush anthocyanins showed two distinct stability profiles: at higher pHs and at lower pHs, with the highest stability being observed in acidic media. The higher pH value decreased the ethanolic anthocyanins storage stability, the half-life ratio values being influenced by the temperature (at 2 °C t_1/2, pH = 3_/t_1/2, pH = 7_ = 1.9 while at 37 °C t_1/2, pH = 3_/t_1/2, pH = 7_ = 2.8 and at 75 °C t_1/2, pH = 3_/t_1/2, pH = 7_ = 3.5).

The storage temperature has a strong influence on the degradation rate of anthocyanins; storage at 37 °C resulted in a faster degradation compared to refrigerated storage at 2 °C, the ratio between the half-life values depending on the pH values (at pH=3, t_1/2, 2 °C_ /t_1/2, 37 °C_ = 10.5, while at pH = 7 the value of this ratio is 15.4). As expected, the increase of the temperature at 75 °C resulted in a more accelerated degradation of the anthocyanins (at pH = 3, t_1/2, 2 °C_/t_1/2, 75 °C_ = 41.4, while at pH = 7 the value of this ratio is 75.7).

According to data summarized in Table 1, at pH = 7, the determined values for the kinetic rate constants and the half-life values during storage at 2 °C and 37 °C are similar to those obtained in the same conditions in the aqueous extract, hence the influence of the storage solvent in these conditions is less important. In contrast, at higher temperatures (such as 75 °C), the solvent manifests a greater influence on the stability of anthocyanin extracts, the degradation rate being 1.8 fold higher in water as in ethanol. 

At pH = 3 and lower temperatures (2 °C and 37 °C), the degradation of anthocyanins present in the ethanolic extract proceeds at twice the rate observed in the aqueous extract. At 75 °C, a slightly faster degradation process of anthocyanins from *Viburnum opulus* *(L.)* fruits was observed in the ethanolic extract, compared to the aqueous one, so one can conclude that in acidic environments and at higher temperatures, the influence of the storage solvent on the stability of monomeric anthocyanins from *Viburnum opulus (L.)* is less important.

To determine the effect of temperature on the kinetics of the degradation process, the constants obtained from Equations (1) and (2) were fitted to an Arrhenius type equation (Equation (3)):


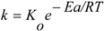
(3)

where E_a_ = the activation energy (kJ/mol); K_o_ = frequency factor (h^−1^); R = the universal gas constant (8.314 J/mol·K); T = absolute temperature (K).

**Figure 3:**
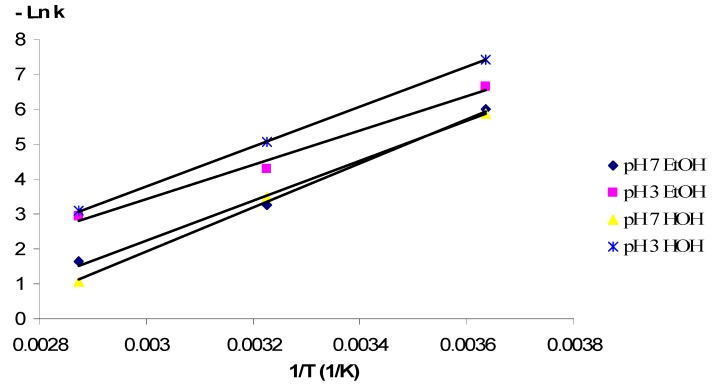
The Arrhenius plots for degradation of anthocyanins in cranberrybush fruits extracts.

The anthocyanin degradation rate constants obtained for each extract were plotted as a function of temperature (Figure 3). The calculated activation energies are given in Table 2.

**Table 2:** Effect of temperature on the degradation of anthocyanins from cranberrybush fruits extracts.

pH	Solvent	E_a_ (kJ/mol)^a^	K_o_ (h^−1^)	Q_10_	
				2-37 °C	37-75 °C
3	Water	47.39 (0.9886)	5.94 × 10^5^	1.018	1.681
7		52.47 (0.9975)	2.43 × 10^7^	1.978	1.898
3	Ethanol/Water	40.79 (0.9890)	8.08 × 10^4^	1.956	1.436
7		47.34 (0.9999)	2.84 × 10^6^	2.184	1.52

^a^ Numbers in parantheses, R^2^, are the determination coefficients.

High activation energy implies that the anthocyanins in the extracts are more susceptible to degradation by exposure to elevated temperatures. The highest influence of the temperature on the degradation process (the highest value of E_a_) was observed for the anthocyanins stored in water at pH = 7, while the lowest value of the E_a_ (lower susceptibility to thermal degradation) was obtained for storage in ethanol at pH = 3. The cross influence of the pH and solvent on the degradation process of the anthocyanins extracted from *Viburnum opulus (L.)* fruits seems to be more significant in case of storage in water at pH = 3 and ethanol at pH = 7 (where the activation energy values are practically identical).

The dependence of degradation rate on temperature was also evaluated by calculating the temperature coefficient Q_10_, according to Equation (4):


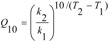
(4)

where Q_10_ = the temperature coefficient (K^−1^); k_1,2_ = rate constant (h^−1^) at temperature T_1,2_ (K).

Almost the same Q_10_ values were obtained for the degradation of anthocyanins in water at pH = 7, proving that the influence of the increase of the temperature on the stability of the anthocyanins is the same for both studied temperature intervals (2–37 °C and 37–75 °C). The lowest temperature coefficient value (1.018 K^−1^ at 2–37 °C) was obtained in water at pH = 3 indicating that low storage temperatures and acidic media are needed to inhibit degradation of anthocyanins from European cranberrybush fruits extracts.

Since no degradation studies were performed on the European cranberrybush fruits anthocyanins, the calculated t_1/2_ values are compared to the literature reported t_1/2_ values for the degradation process of anthocyanins obtained from other sources. The t_1/2_ values were reported to be 55.7 days (at 4 °C) and 2.1 days (at 37 °C) in blood orange juice concentrate [26] and 22.1 days and 1.15 days, respectively, for black carrot anthocyanins [22]. Compared to these values, our results are in the same range (t_1/2_ = 48.1 days at 2 °C and 4.5 days at 37 °C, respectively), differences being due to the different storage pH values. At higher temperature values (80 °C and pH = 3), t_1/2_ = 34 h for red flesh potato, 33 h for purple carrot and 15 h for grapes anthocyanins were reported [27]. These results indicate that anthocyanins from European cranberrybush fruits are less stable at higher temperatures (t_1/2_ = 15.03 h) than those obtained from red flesh potato and purple carrot.

## 3. Experimental

### 3.1. Plant Material

Samples of European cranberrybush fruits were harvested in October 2010 from Cluj-Napoca, Romania. Fruits were packed in polyethylene bags and kept frozen at −18 °C before being subjected to anthocyanin extraction.

### 3.2. Chemicals and Reagents

All chemicals and reagents were of analytical grade and were purchased from Merck Germany and were used without further purification.

### 3.3. Preparation of Ethanolic and Aqueous Anthocyanin Extracts

The frozen cranberrybush fruits were crushed in a mortar. Five g of fruits were transferred to an Erlenmeyer flask and solvent (200 mL, water or 96% ethanol) and concentrated HCl (0.25 mL) were added. The mixture was stirred for 1 h at room temperature and then filtered through Whatman no. 1 paper under vacuum. The resulted plant material was reextracted twice with acidified solvent (20 mL) until a faint-colored extract was obtained. The filtrates were quantitatively transferred to a 250 mL volumetric flask and made up to the mark with the appropriate solvent. The crude extract was used to measure the total anthocyanin content and to investigate the effect of pH, temperature and solvent on the stability of European cranberrybush anthocyanins. Extractions repeated on three independent fruit samples using 0.1% HCl in ethanol 96% (v:v) as solvent were completed in order to determine the mean value of total anthocyanin content/kg frozen fruits.

### 3.4. Determination of Anthocyanin Content

The total pigment concentration was measured by the easy and convenient method of Giusti and Wrolstad [25], based on the structural changes of their chemical forms as a function of pH, which can be measured using optical spectroscopy. 

The red to purple oxonium form predominates at pH = 1, while at pH = 4.5, the colorless hemiketal form is the major structural form. The difference in absorbance of the anthocyanins solutions between these two pH values, permits an accurate and rapid determination of total monomeric anthocyanins content in the sample matrix.

The pH-differential method [25] was used to determine the total anthocyanin content, using two buffer systems: potassium chloride buffer (0.025 M, pH = 1.0) and sodium acetate buffer (0.04 M; pH = 4.5). The crude fruit extract was mixed with the corresponding buffer and the absorbance of the samples was measured at 525 nm (λ_VIS max_) and 700 nm.

Aliquots of the extract (5 mL) were transferred to a 10 mL volumetric flask and made up to 10 mL with corresponding buffer (pH = 1 and pH = 4.5) and allowed to equilibrate for 15 minutes. Three identical samples were prepared for each pH value and anthocyanin content was measured one time for each replicate.

The absorbance of each equilibrated solution was then measured at 525 (λ_VIS max_) and 700 nm (for haze correction), using an UV-VIS Perkin Elmer Lambda 35 double beam spectrophotometer.

Pigment content was calculated as equivalents of cyanidin-3-glucoside (MW = 449.2 g/mol, ε = 26,900 L/mol/cm). Visible spectra of samples were recorded by scanning the absorbance between 400 and 700 nm. Quartz cuvettes of 1 cm pathlength were used. Absorbance readings were made against distilled water as a blank. 

From the experimental data, the total anthocyanin content (as cyanidin-3-glucoside equivalents), was calculated using the following equation: 


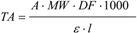
(5)

where TA = total anthocyanin content (mg/L); MW = molecular weight; DF = dilution factor; l = pathlength; ε = molar extinction coefficient; 1,000 = conversion factor from gram to milligram. 

A = absorbance, calculated by Equation (6):

A = (A_pH 1.0_ − A_pH 4.5_)_ 525 nm_ − (A_pH 1.0_ − A_pH 4.5_) _700 nm_ (6)

All the measurements were done in triplicate (n = 3), at room temperature (~22 °C) and the obtained mean values of total anthocyanin content were used for kinetic parameters determination of anthocyanin degradation.

### 3.5. Degradation Studies

The influence of different factors (solvent, temperature and pH) on the stability of anthocyanins from the Cranberrybush fruits extract during storage was studied. The effect of pH on storage stability was studied at two different pH values (3.0 and 7.0). To obtain the desired pH values for the samples, the extract was homogenized with pH = 3 acetate buffer and pH = 7 phosphate buffer respectively (1:1 v/v). The pH values were verified by pH-meter measurements.

The influence of temperature on the stability of anthocyanins was evaluated for both aqueous and ethanolic extracts at pH = 3 and pH = 7 at three different temperatures (2 °C, 37 °C and 75 °C). Aliquots of 50 mL of each buffered extract (pH3-EtOH, pH7-EtOH, pH3-HOH, pH7-HOH) were put into screw-cap test tubes, kept away from light at 2 °C (in refrigerator) and in a previously equilibrated thermostatic water bath, at 37 °C and 75 °C, respectively (±1 °C). At regular time intervals, samples were removed from the refrigerator and water baths and the analyses were conducted immediately.

Measurements were taken at time 0 h, 12 h, 1 day, 2 days, 3 days, 4 days, 5 days, 6 days and 1 week for all extracts, except for those stored 3 days or less (see table 3) that were sampled at 0 h, 2 h, 4 h, 6 h, 8 h, 10 h, 12 h, 14 h, 1 day, 2 days, 3 days.

**Table 3:** Total storage times of anthocyanins from cranberrybush fruits extracts in different conditions.

pH	Temp. (°C)	Solvent	Total storage time (h)
3	2	Water	167
3	37	Water	48
3	75	Water	72
7	2	Water	167
7	37	Water	14
7	75	Water	12
3	2	Ethanol/Water	167
3	37	Ethanol/Water	145
3	75	Ethanol/Water	72
7	2	Ethanol/Water	145
7	37	Ethanol/Water	97
7	75	Ethanol/Water	14

The storage periods were different for each thermal treatment, due to differences in anthocyanin degradation rates. The stability of the extracts was evaluated by measuring changes in total anthocyanins content of the samples.

### 3.6. Data Analysis

Data are reported as mean values of triplicate experiments. Results were processed using one-way variance analysis (ANOVA). Differences at *p* < 0.05 were considered statistically significant. 

## 4. Conclusions

The results from the present study have provided detailed information regarding the stability of anthocyanins in European cranberrybush fruit extracts, which was strongly dependent on temperature, pH and storage solvent. Increasing pH and temperature during heating and storage increased the degradation rate constants of the investigated anthocyanins. The degradation rate proved to be slightly dependent on the nature of the storage solvent. At pH = 7 (at 2 °C and 75 °C) the degradation process was faster in water compared to ethanol. In all the other investigated storage conditions, the stability of anthocyanins was higher in water.

In order to keep the anthocyanin degradation rate as low as possible, it is recommended that European cranberrybush anthocyanin extracts should be kept at refrigeration temperatures, in a aqueous, slightly acidic environment (pH = 2–3).

The study of the degradation changes of anthocyanin extracts from European cranberrybush at various storage temperatures, solvents and pH values, supports the potential use of these berries as a source of natural red to purple pigments for the food industry.
